# Adiposity significantly modifies genetic risk for dyslipidemia[S]

**DOI:** 10.1194/jlr.P052522

**Published:** 2014-11

**Authors:** Christopher B. Cole, Majid Nikpay, Paulina Lau, Alexandre F. R. Stewart, Robert W. Davies, George A. Wells, Robert Dent, Ruth McPherson

**Affiliations:** * Atherogenomics Laboratory, University of Ottawa Heart Institute, Ottawa, Canada; † Ruddy Cardiovascular Genetics Centre, University of Ottawa Heart Institute, Ottawa, Canada; § Cardiovascular Research Methods Centre, University of Ottawa Heart Institute, Ottawa, Canada; ** Bariatric Centre of Excellence, Ottawa Hospital, Ottawa, Canada

**Keywords:** obesity, genetic risk score, lipoproteins, single nucleotide polymorphism, statistical interaction

## Abstract

Recent genome-wide association studies have identified multiple loci robustly associated with plasma lipids, which also contribute to extreme lipid phenotypes. However, these common genetic variants explain <12% of variation in lipid traits. Adiposity is also an important determinant of plasma lipoproteins, particularly plasma TGs and HDL cholesterol (HDLc) concentrations. Thus, interactions between genes and clinical phenotypes may contribute to this unexplained heritability. We have applied a weighted genetic risk score (GRS) for both plasma TGs and HDLc in two large cohorts at the extremes of BMI. Both BMI and GRS were strongly associated with these lipid traits. A significant interaction between obese/lean status and GRS was noted for each of TG (*P_Interaction_* = 2.87 × 10^−4^) and HDLc (*P_Interaction_* = 1.05 × 10^−3^). These interactions were largely driven by SNPs tagging *APOA5*, glucokinase receptor (*GCKR*), and *LPL* for TG, and cholesteryl ester transfer protein (*CETP*), GalNAc-transferase (*GALNT2*), endothelial lipase (*LIPG*), and phospholipid transfer protein (*PLTP*) for HDLc. In contrast, the GRS_LDL cholesterol_ × adiposity interaction was not significant. Sexual dimorphism was evident for the GRS_HDL_ on HDLc in obese (*P_Interaction_* = 0.016) but not lean subjects. SNP by BMI interactions may provide biological insight into specific genetic associations and missing heritability.

Recent genome-wide association studies (GWASs) have identified multiple genetic variants robustly associated with plasma lipid traits. The Global Lipids Consortium reported 157 significant loci (*P* < 5 × 10^−8^) (1, 2). Many are novel, and several encompass genes not previously implicated in plasma lipid metabolism. Furthermore, these loci were shown to contribute not only to general variation in plasma lipids, but also to extreme lipid phenotypes (3). Notably, for TGs, individuals in the top quartile of the TG risk score were 44 times more likely to have hypertriglyceridemia as compared with individuals in the bottom quartile (*P* = 4 × 10^−28^). For HDL cholesterol (HDLc), individuals in the top quartile of the risk score were four times more likely to have high HDLc as compared with those in the bottom quartile (1).

Although family-based association studies indicate that 40% to 60% of variation in plasma TG and HDLc is genetically based (4, 5), the identified loci explain <12% of variation in each of these lipid traits (1). Environmental and clinical factors including BMI, physical activity, and alcohol intake are also important determinants of plasma TG and HDLc (6).

Thus, interactions between genetic risk factors and clinical phenotypes may account for some of the unexplained heritability of plasma lipid traits. Here we have examined whether the effect of a weighted genetic risk score (GRS) on each of TG and HDLc is modified by adiposity, as assessed by BMI. This study provides biological insight into specific genetic associations and may aid in the identification of dyslipidemic subjects for whom weight loss is likely to be an important intervention.

## METHODS

### Study subjects

Subjects with a BMI ≥30 kg/m^2^ were defined as obese, those with a BMI ≤23 kg/m^2^ as lean, and intermediate subjects (30 kg/m^2^ ≥ BMI ≥ 23 kg/m^2^) as normal range. The BMI cutoff of ≤23 for the lean subgroup is below the 25th percentile for the majority of individuals studied. Two cohorts were studied.

#### Obese versus lean.

Obese, unrelated subjects of strictly European ancestry were recruited from the University of Ottawa Weight Management Clinic. Obese individuals displayed a BMI of >35 kg/m^2^ and a history of at least 10 years of adult obesity with no medical or psychiatric predisposing factors. Unrelated lean subjects were recruited from the Ottawa community. These healthy individuals had a lifelong BMI of less than the 25th percentile for sex and age, and no medical or psychiatric conditions affecting body weight (7, 8). Body weight was measured using a Tanita electronic scale to the nearest 0.3 kg. BMI was defined as weight in kilograms divided by height in meters squared (kg/m^2^). Height was measured to the nearest 0.5 cm. Plasma lipid fractions were measured using standard procedures. For coronary artery disease controls (CAD-C) subjects on lipid modifying medication, written documentation of pretreatment plasma lipids was obtained from the primary care physician and used for these analyses. These data were not available for 6.4% of the CAD-C subjects, none of whom were treated with a fibrate or niacin. In the obese versus lean (OBLE) cohort, 2.6% of lean and 14.8% of obese subjects were on low- to moderate-dose statin therapy, not expected to have major effects on TG or HDLc. The study was approved by the Human Ethics Experimentation Committees of the University of Ottawa Heart Institute and the Ottawa Hospital and written informed consent was obtained from all subjects.

#### CAD-C.

Details of the CAD-C cohorts have been previously described (9). Briefly, CAD-C included healthy controls recruited as part of the Ottawa Heart Genomics Study in collaboration with the Cleveland Clinic Gene Bank (OHGS_A and OHGS_CCGB_B). These subsets were combined together to form a single CAD-C sample. Subjects were collected under human research protocols approved by their respective committees.

### Genotyping and imputation

SNP genotyping of the OBLE and CAD-C cohorts was performed on Affymetrix 6.0 or 500K Arrays at the University of Ottawa Heart Institute using the standard protocol recommended by the manufacturer and processed as described (10, 11). Imputation was performed using IMPUTE2 and the August 2009 1000 Genomes European reference panel (12). After imputation, ∼5.5 M SNPs passed post-quality control (QC) measures (info >0.5, Hardy Weinberg Equilibrium >1e–6, missing <10%).

### Selection of GWAS SNPs

To create weighted GRSs for TG (GRS_TG_) and HDLc (GRS_HDLc_), we applied the findings of the Global Lipids Consortium 2010 study, which performed a fixed-effects meta-analysis on 46 separate GWASs comprising >100,000 individuals of European descent at a total of ∼2.6 million imputed or directly genotyped (1). Because the Global Lipids SNPs were identified in populations separate from those being considered here, we have avoided the bias inherent in performing discovery and effect size estimation in the same data set.

### GRS

SNPs were individually coded as 0, 1, or 2, according to the number of trait-increasing alleles at that particular SNP. To generate the GRS_TG_, 20 SNPs were analyzed in the population; to generate the GRS_HDL_, 34 SNPs were analyzed. To generate GRS for LDL cholesterol (GRS_LDLc_), 11 SNPs were analyzed. Several SNPs for each trait failed to pass QC in our populations and were thus excluded from analysis. If a particular SNP failed QC in a particular subgroup, it was coded as missing in the total population. A weighted GRS (*Ŝ*) was constructed for each individual by taking a sum across SNPs of the number of reference alleles (0, 1, or 2) at that SNP and multiplying by the β effect score of that allele. Thus, we define **G** as an *m-*vector of coded markers (0, 1, or 2) and β as the effect size at that allele defined by the Global Lipids Consortium (1, 13, 14).

$$\hat{S}=\frac{\sum_{i=1}^{m}\beta_{i}G_{i}}{m}$$

After experimentation with various methodologies, we concluded that a weighted GRS outperforms allele counting or a merely additive model (9, 13, 15). GRSs were constructed in PLINK: whole-genome association analysis toolset (14). SNPs and corresponding effect sizes for each of TG and HDLc are provided in supplementary Table I. Effect sizes provided are for the primary trait only.

### Statistical analysis

Individual post-QC genotyped SNPs were coded as 0, 1, or 2 according to the number of effect alleles present, and a weighted GRS was constructed for each individual according to the previously described procedure for each of TG and HDLc. Multiple general linear regression models (GLMs) were used to test for the association between genotypes and HDLc and TGs. Data were adjusted for age, sex, and age^2^. Response data were broken down into lean, obese and normal range categories in order to investigate the effect of genetic risk across the BMI spectrum. Each SNP was tested for associations to phenotype separately from the GRS using GLMs, and interaction scores were constructed for SNP × obese/lean status and SNP × sex by including an interaction term in the respective models. The same covariates, which were used to analyze the data, were also controlled for when determining SNP × obese/lean status and SNP × sex interaction terms. Data were further stratified by gender. All analyses were conducted in PLINK (14) and R version 3.0.0 (http://www.r-project.org/).

## RESULTS

The general characteristics of obese and lean subjects in each of the two main cohorts are shown in **Table 1**. Within the OBLE and CAD-C cohorts, subjects were well matched for age and sex. The OBLE cohort was younger and exhibited greater extremes of BMI [mean 43.1 ± 0.3 (obese); 20.3 ± 0.1 kg/m^2^ (lean)] as compared with the CAD-C group [mean BMI 34.6 ± 0.2 (obese); 21.3 ± 0.1 kg/m^2^ (lean)].

**TABLE 1.** Characteristics of the study sample separated by cohort and by trait under study

	n	Male (%)	Age (years)	BMI (kg/m^2^^2^)	Risk Score
OBLE					
TG					
n	1,784	34.1	45.4 ± 0.3	31.8 ± 0.3	−0.198 ± 0.01
Lean	868	39.4	44.5 ± 0.5	20.3 ± 0.1	−0.192 ± 0.014
Obese	916	28.9	46.4 ± 0.4	43.1 ± 0.3	−0.204 ± 0.013
HDL					
n	1,779	34.3	45.5 ± 0.3	31.7 ± 0.3	0.007 ± 0.001
Lean	868	39.4	44.5 ± 0.5	20.3 ± 0.1	0.009 ± 0.002
Obese	911	25.1	46.4 ± 0.4	43.0 ± 0.3	0.005 ± 0.002
CAD-C					
TG					
n	2,966	49.4	75 ± 0.1	26.3 ± 0.1	−0.149 ± 0.006
Lean	788	46.4	75.8 ± 0.2	21.6 ± 0.1	−0.142 ± 0.011
Obese	338	37.1	73.4 ± 0.2	34.6 ± 0.2	−0.145 ± 0.016
Normal	1,840	55.2	74.9 ± 0.1	26.8 ± 0.1	−0.153 ± 0.007
HDL					
n	2,937	49.3	74.9 ± 0.1	26.3 ± 0.1	−0.006 ± 0.001
Lean	596	48.2	76.1 ± 0.2	21.1 ± 0.1	−0.006 ± 0.002
Obese	498	32.6	73.9 ± 0.2	33.4 ± 0.1	−0.005 ± 0.002
Normal	1,843	55.0	74.8 ± 0.1	26.1 ± 0.1	−0.005 ± 0.001
Total					
TG					
n	4,718	43.7	63.9 ± 0.2	28.4 ± 0.1	−0.167 ± 0.005
Lean	1,656	38.3	59.4 ± 0.5	20.9 ± 0.1	−0.168 ± 0.009
Obese	1,222	33.7	53.8 ± 0.4	40.7 ± 0.2	−0.188 ± 0.011
Normal	1,840	55.3	74.9 ± 0.1	26.8 ± 0.1	−0.153 ± 0.007
HDL					
n	4,683	43.6	63.8 ± 0.2	28.3 ± 0.1	−0.001 ± 0.001
Lean	1,464	36.6	57.3 ± 0.5	20.6 ± 0.1	0.003 ± 0.001
Obese	1,376	35.6	56.3 ± 0.4	39.5 ± 0.2	0.001 ± 0.001
Normal	1,843	55.0	74.8 ± 0.1	26.1 ± 0.1	−0.005 ± 0.001

Values represent mean ± standard deviation, unless otherwise indicated. Lean: BMI <23 kg/m^2^ and less than 25th percentile. Obese: BMI >30 kg/m^2^ for >10 years. Normal: 23 kg/m^2^ ≤ BMI ≤ 30 kg/m^2^. Risk score corresponds to the sum of the effect size per risk gene multiplied by the effect size of that risk gene, divided by the total number of risk genes. Data are provided as mean ± standard deviation. See supplementary Table I for further details.

For the entire group, the mean difference in TG for subjects above or below the 50th percentile of the weighted GRS_TG_ was 0.191 mM [95% confidence interval (CI) = 0.140–0.241, *P* = 1.92 × 10^−13^]. For obese subjects, this difference was 0.325 mM (95% CI = 0.250–0.399, *P* < 2.20 × 10^−16^) and for lean subjects 0.114 mM (95% CI = 0.250–0.399, *P* < 2.20 × 10^−16^). The mean difference in HDLc for all subjects above or below the 50th percentile of the GRS_HDL_ (based on HDLc-raising alleles) was 0.129 mM (95% CI = 0.106–0.153, *P* < 2.2 × 10^−16^). This value was lower for the obese (0.108 mM; 95% CI = 0.075–0.141, *P* = 2.25 × 10^−10^) and higher for the lean (0.166 mM; 95% CI = 0.124–0.208, *P* = 1.97 × 10^−14^) subjects.

**Fig. 1.**
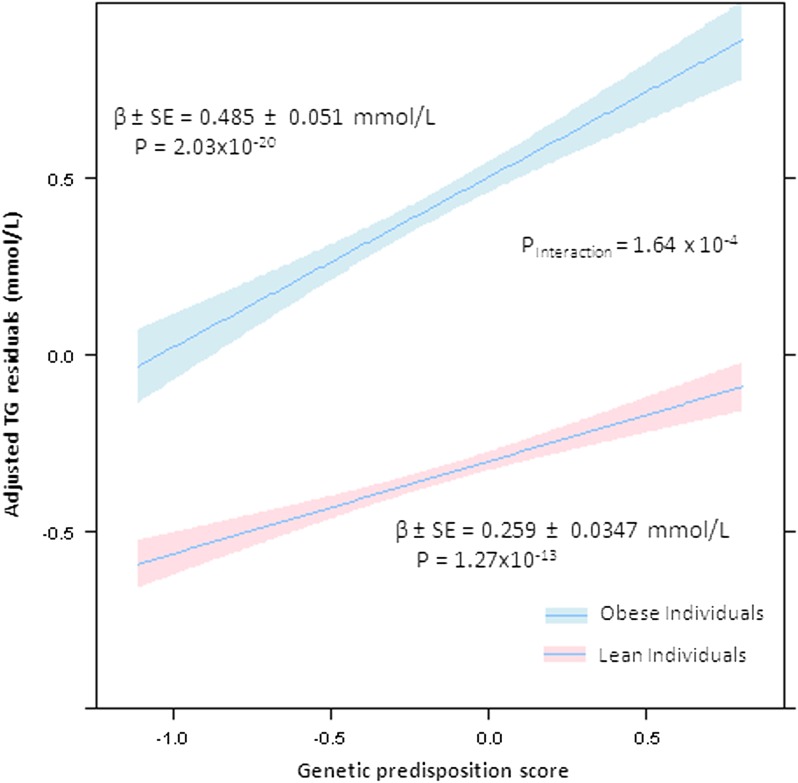
TG residuals compared with GRS stratified by lean versus obese status. Significantly differently slope coefficients with 95% CIs are displayed, demonstrating a significant interaction between obesity status and a GRS. The rate of increased TG residuals for an increased predisposition is displayed for obese (broken line) and lean (solid line) individuals. Increased risk in obese individuals corresponds to an increased expression of lipid levels above what would normally be expected. This dimorphic effect was dependent on three SNPs tagging *APOA5*, glucokinase receptor (*GCKR*), and *LPL*, not before observed to have adiposity-dependent dimorphic effects.

**Fig. 2.**
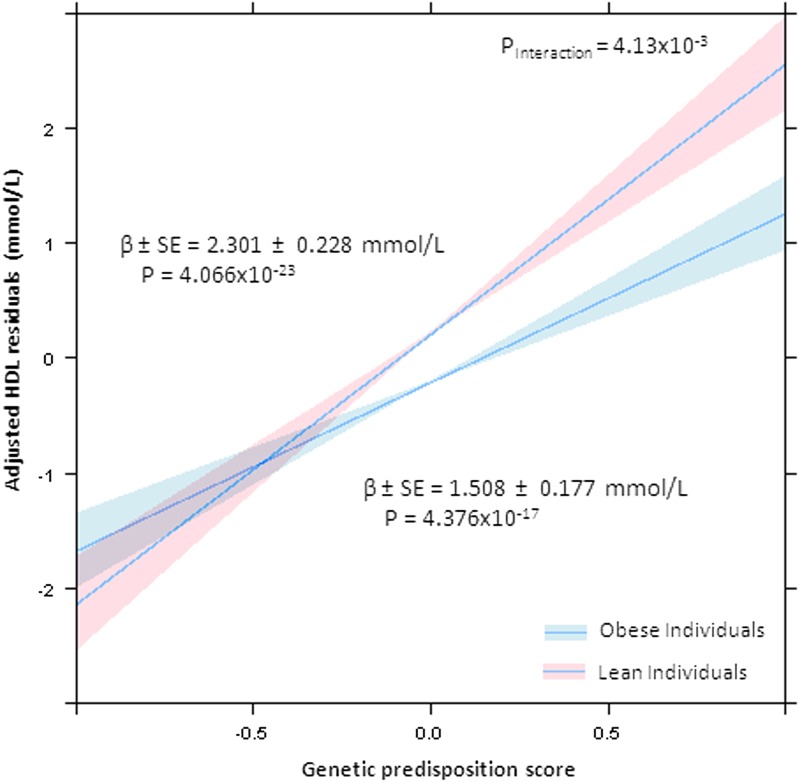
HDLc residuals compared with GRS stratified by lean versus obese status. Significantly differing slope coefficients with 95% CIs are displayed for obese and lean populations. Lean individuals exhibit a greater response to a larger number of HDLc-raising alleles. The dimorphic effect in HDL is due to SNPs tagging cholesteryl ester transfer protein (*CETP*), endothelial lipase (*LIPG*), GalNAc-transferase (*GALNT2*), and phospholipid transfer protein (*PLTP*), loci not previously noted to exhibit adiposity-dependent dimorphism.

As shown in **Table 2**, subsequent analysis by covariate adjusted multiple linear models revealed a significant difference in the effect size (β) of the GRS on each of TG and HDLc in the obese versus lean subgroups. For GRS_TG_ on TG in the obese population, β = 0.480 mM (SE = 0.0533, *P* = 8.97 × 10^−19^), versus for the lean subgroup, β = 0.261 mM (SE = 0.0336, *P* = 1.52 × 10^−14^), with a significant interaction term (*P_Interaction_ =* 2.87 × 10^−4^) (**Fig. 1**). For GRS_HDL_ and HDLc in the obese sample, β = 1.466 mM (SE = 0.166, *P* = 2.49 × 10^−18^) versus β = 2.347 mM (SE = 0.209, *P* = 3.41 × 10^−28^) in the lean, demonstrating significant interactions for obese/lean status × GRS_HDL_ (*P_Interaction_* = 1.05 × 10^−3^) (**Fig. 2**). For GRS_LDLc_ in the obese population, β = 0.434 mM (SE = 0.0831, *P* = 2.14 × 10^−7^), similar to the lean population where β = 0.390 mM (SE = 0.0715, *P* = 5.63 × 10^−8^). As expected, no significant interaction between GRS_LDLc_ and obese/lean status was found (*P_Interaction_* = 0.689). Subjects with a BMI in the normal range (23 kg/m^2^ < BMI < 30 kg/m^2^) exhibited a value between the lean and obese for TG, β = 0.354 mM (SE = 0.0289, *P* = 4.68 × 10^−34^); for HDLc, β = 1.91 mM (SE = 0.126, *P* = 2.16 × 10^−50^); but not for LDL, β = 0.464 mM (SE = 0.0473, *P* = 1.54 × 10^−22^). Subjects with a BMI in the normal range (23 kg/m^2^ < BMI < 30 kg/m^2^) exhibited a value between the lean and obese for TG, β = 0.354 mM (SE = 0.0289, *P* = 4.68 × 10^−34^); for HDLc, β = 1.91 mM (SE = 0.126, *P* = 2.16 × 10^−50^); but not for LDL, β = 0.464 (SE = 0.0473, *P* = 1.54 × 10^−22^).

**TABLE 2.** Associations of GRS with adjusted lipid trait stratified by adiposity

	Obese				Lean				
Trait	n*^a^*	β*^b^* (SE)	*P*	*R*^2^	n	β (SE)	*P*	*R*^2^	*P_Interaction_*
TG	1,222	0.480 (0.053)	8.98E–19	0.0614	1,656	0.261 (0.034)	1.52E–14	0.0345	0.000287
HDL	1,376	1.466 (0.165)	2.49E–18	0.0533	1,464	2.347 (0.209)	3.41E–28	0.0790	0.00105

a Number of nonmissing individuals with complete information included in analysis.

b β coefficient for regression, measured in mM.

Because obesity status significantly influenced the clinical expression of these lipid trait loci, we determined the explained variance (*R*^2^) of the GRS_TG_ and GRS_HDLc_ in obese versus lean subjects. For GRS_TG_ on TG, *R*^2^ = 0.0614 for obese versus *R*^2^ = 0.0345 for lean subjects, a 2-fold difference. An opposite trend was observed for GRS_HDLc_ (based on HDLc-raising alleles) on HDLc, *R*^2^ = 0.0790 for lean versus *R*^2^ = 0.0533 for obese. In contrast, for the GRS_LDLc_ on LDLc, explained variance was only slightly higher in the obese (*R*^2^ = 0.0215) versus lean (*R*^2^ = 0.0172) populations.

We next examined the individual SNPs included in the GRS_TG_ and GRS_HDLc_. Three TG SNPs (*APOA5*,* GCKR*, and *LPL*) and four HDLc SNPs (*CETP*,* LIPG*,* GALNT2*, and* PLTP*) were found to have a significant obese/lean status × SNP effect interaction term at a false discovery rate (FDR) of 20% (**Table 3**). *LPL* and *APOA5* achieved a 10% FDR for TG, and *CETP*,* GALNT2*, and *LIPG* reached a 10% FDR for HDLc. However, at a 5% FDR, only *LPL* and *CETP* were significant. Statistical correction for multiple testing was achieved by ordering each tested SNP from least to greatest *P_Interaction_* value. The largest interaction term that was less than the *P_FDR_* [i.e., the ratio of the position of the SNP (i) divided by the number of SNPs analyzed (number of tests performed) multiplied by the FDR] was determined to be the cutoff at which results were classified as significant (16). Further details regarding SNP × obese/lean status analyses are provided in supplementary Table II. To test whether these SNPs were the major contributors to the overall obese/lean status × GRS interaction, a new score was constructed for each group omitting these SNPs. As expected, the interaction term was no longer significant (TG: *P_Interaction_* = 0.196; HDLc: *P_Interaction_* = 0.321).

**TABLE 3.** Individual loci that exert differing effects in obese versus lean subjects

				Obese			Lean			Interaction			
Locus	Lead SNP	Allele*^a^*	Trait	n*^b^*	β (SE)*^c^*	*P*	n	β (SE)	*P*	n	β (SE)	*P_Interaction_*	*^d^*
*LPL*	rs12678919	G	TG	945	–0.148 (0.03)	4.03E–06	932	–0.050 (0.03)	1.13E–01	1,877	–0.21 (0.06)	6.99E–04	0.01
*APOA5*	rs964184	G	TG	1,078	0.159 (0.03)	1.31E–07	1,282	0.14 (0.03)	1.91E–07	2,360	0.15 (0.06)	8.87E–03	0.02
*GCKR*	rs1260326	T	TG	1,189	0.0932 (0.03)	1.21E–03	1,569	0.067 (0.02)	6.84E–03	2,758	0.12 (0.05)	2.82E–02	0.03
*CETP*	rs3764261	A	HDL	1,083	0.132 (0.03)	1.67E–06	1,238	0.189 (0.03)	3.15E–13	2,415	–0.21 (0.05)	1.14E–05	0.005882
*GALNT2*	rs4846914	G	HDL	1,212	–0.065 (0.03)	1.24E–02	1,463	–0.002 (0.02)	9.39E–01	2,829	–0.13 (0.05)	3.03E–03	0.011765
*LIPG*	rs7241918	G	HDL	1,061	–0.004 (0.03)	8.96E–01	1,178	–0.102 (0.03)	1.35E–04	2,329	0.13 (0.05)	7.00E–03	0.017647
*PLTP*	rs6065906	C	HDL	1,184	–0.034 (0.03)	2.01E–01	1,435	–0.107 (0.02)	1.19E–05	2,769	0.10 (0.04)	2.08E–02	0.023529

a Active allele analyzed.

b Number of nonmissing individuals with complete information used in analysis.

c β coefficient for regression; measured in mM (standard error).

d FDR of 20% displayed. All achieved FDR <20%; *APOA5*,* GALNT2*, and *LIPG* achieved FDR <10%; *LPL* and *CETP* achieved FDR <5%.

### Sex × lipid trait interactions

Next, we investigated whether GRS effects differed by sex. Of note, sex did not significantly influence the effect of the GRS on any trait in the whole population (TG: *P_Interaction_* = 0.0925; HDLc: *P_Interaction_* = 0.0868; LDL: *P_Interaction_* = 0.189). However, for GRS_HDLc_ on HDLc, there was a significant interaction with sex in the obese (*P_Interaction_* = 0.016) but not the lean (*P_Interaction_* = 0.369) population. A sex dimorphic effect by obese/lean stratification was not found for the other lipid traits. Further analysis of individual SNPs failed to identify significant interaction terms in the whole population for either TG or HDLc. However, one sexually dimorphic locus for HDLc was found in each of the lean (rs4846914 tagging *GALNT2*) and obese [rs605066 tagging Cbp/p300-interacting transactivator, with Glu/Asp-rich carboxy-terminal domain, 2 (*CITED2*)] populations. However, after correction for multiple testing, these loci were only nominally significant (FDR = 15%) (**Table 4**). More complete SNP × sex interaction data may be found in supplementary Table III.

**TABLE 4.** Individual SNPs with suggestive evidence of sexual dimorphism

				Male			Female			Interaction			
Locus	Lead SNP	Allele*^a^*	Pop*^b^*	n*^c^*	β (SE)*^d^*	*P*	n	β (SE)	*P*	n	β (SE)	*P_Interaction_*	*^e^*
*GALNT2*	rs4846914	G	OB	405	0.060 (0.05)	2.15E–01	807	–0.12 (0.03)	3.97E–04	1,212	0.14(0.04)	2.09E–03	4.84E–03
*CITED2*	rs605066	C	LE	515	0.694 (0.29)	1.75E–02	894	–0.058 (0.03)	7.77E–02	1,409	0.12(0.04)	4.57E–03	4.84E–03

a Active allele analyzed.

b Population where loci are active.

c Number of nonmissing individuals with complete information used in analysis.

d β coefficient for regression; measured in mM (standard error).

e FDR of 15% displayed. *GALNT2* is significant at <10% FDR.

## DISCUSSION

Lifestyle and clinical factors may modify genetic risk. For example, the effect of a GRS on BMI was found to be significantly attenuated in physically active versus sedentary individuals (17). To explore the effects of adiposity on genetic risk for dyslipidemia, we have utilized a GRS constructed from loci previously reported by the Global Lipids Consortium. We demonstrate that obesity status significantly alters the effect of genetic variants associated with increased TGs as well as those associated with higher levels of HDLc, but not LDLc.

For TG, the effect size (β) of a weighted GRS_TG_ in the obese population was nearly double that of the lean population (β = 0.480 vs. 0.261). As shown in Fig. 1, for any GRS_TG_, plasma TG levels are greater for obese versus lean subjects. This is not surprising given known effects of substrate availability on hepatic TG synthesis; in obese individuals, the effect of nutrient excess outweighs the effect of known genetic variants at any GRS_TG_. In both obese and lean individuals, GRS_TG_ associates with higher TGs, but the slope of the line for GRS_TG_ versus TG differs for obese as compared with lean yielding a significant interaction coefficient. Overall, the variance in plasma TG concentrations explained by the GRS_TG_ was 6.14% for the obese subjects, nearly double that found for the lean population (3.45%).

In contrast to TGs, the effect of a GRS_HDL_, consisting of HDLc-raising alleles, on HDLc was greater for the lean (β = 2.347) than the obese (β = 1.466) population. For HDLc, it is important to note that we created a GRS_HDL_ composed of HDLc-raising alleles (a genetic protective score for HDL). As shown, obese individuals have higher circulating levels of TG-rich lipoproteins, leading to TG enrichment of HDL and more rapid HDL clearance. Thus, as shown in Fig. 2, it is likely that the metabolic effect of hypertriglyceridemia acts to attenuate the effect of HDL-raising alleles, for example near genes encoding CETP, LIPG, and PLTP. The GRS_HDL_ for HDLc explained 7.89% of HDLc variation in the lean versus 5.33% in the obese subjects.

Thus, the genetic risk for hypertriglyceridemia is significantly worsened by the obese state, whereas the beneficial effect of HDLc-raising genetic variants is attenuated. These data demonstrate that the gene × adiposity interaction contributes to part of the hitherto unexplained genetic variance in plasma lipids levels.

Here, we utilized an aggregated, weighted risk score rather than the more common allele counting method. In the past, allele counting, also known as an additive model, has been used due to a lack of well-established effect sizes (14). However, a weighted, aggregated risk score has been shown to improve power (7, 10). We did not perform receiver operating characteristic area-under-curve analysis because hypertriglyceridemia (high TG) and hypoalphalipoproteinemia (low HDL) are defined by age- and sex-dependent quantiles.

Although we lack the statistical power necessary to detect the individual effects of all loci, we identified seven novel loci not previously reported to have obesity-related dimorphic effects. SNPs tagging *APOA5* (*P_Interaction_* = 8.87 × 10^−3^), *GCKR* (*P_Interaction_* = 2.82 × 10^−2^), and *LPL* (*P_Interaction_* = 6.69 × 10^−4^) showed interaction with obese/lean status for TG. These encompass genes encoding proteins altering both hepatic TG synthesis and peripheral lipolysis. The *GCKR* gene product, the glucokinase regulatory protein, regulates glucokinase (GCK) activity competitively with respect to the substrate glucose, inhibiting GCK activity. Hepatic GCK activity enhances glycolytic flux, promoting hepatic glucose metabolism and increasing malonyl CoA availability, a major substrate for de novo hepatic lipogenesis (18). *LPL* and *APOA5* encode major determinants of peripheral lipolysis of TG-rich lipoproteins, LPL and ApoA5, the latter a regulator of LPL activity (19). The effect sizes of the previously discussed TG loci were among the highest in this study (*APOA5*_β_ = 16.95, *GCKR*_β_ = 8.76, and *LPL*_β_ = –13.64) and not surprisingly were responsible for the significant obese/lean status × GRS interaction. Consistently, in a Filipino population the *APOA5* effect on plasma TG levels was found to be modified by waist circumference (20), another measure of adiposity.

For HDLc, interactions were noted for SNPs tagging *CETP* (*P_Interaction_* = 1.14 × 10^−5^), *LIPG* (*P_Interaction_* = 7.00 × 10^−3^), *GALNT2* (*P_Interaction_* = 3.03 × 10^−3^), and *PLTP* (*P_Interaction_* = 2.08 × 10^−2^). The roles of CETP, LIPG, and PLTP in HDL remodeling in the intravascular space are well known. *GALNT2* encodes GalNAc-transferase believed to play a critical role in *O*-glycosylation of proteins involved in lipid metabolism, including angiopoietin-like 3 (21). In the mouse, altered hepatic *GALNT2* expression significantly modifies circulating HDLc levels (1). Although these HDLc loci exhibited lower effect sizes (*CETP*_β_ = 3.39, *LIPG*_β_ = –1.31, *PLTP*_β_ = –0.93, and *GALNT2*_β_ = –0.61) as compared with the top TG SNPs, they were similarly responsible for the significant GRS_HDLc_ × obese/lean status interaction term. In contrast, no significant interaction was found for GRS_LDLc_ × obese/lean status.

**Fig. 3.**
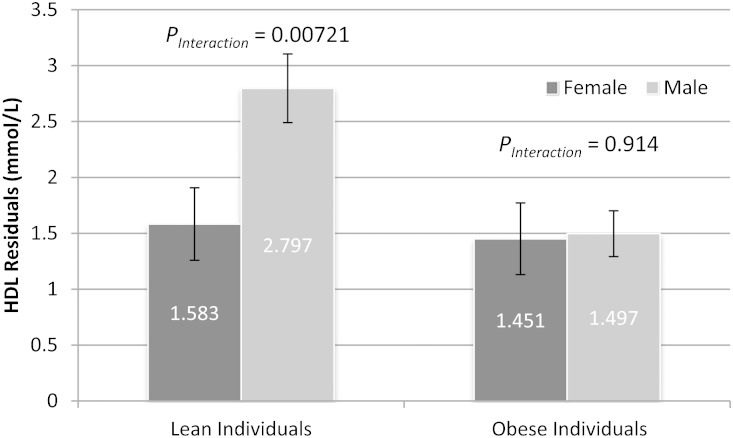
Regression coefficients for HDLc (mM) for male versus female subjects stratified by lean versus obese status. Data are shown for males and females stratified by adiposity, and bars represent SE. In the lean population, women and men display a similar response to GRS_HDLc_. In contrast, obese men demonstrate an attenuated effect of GRS_HDLc_ as compared with obese women. One locus was found to be exhibit sexually dimorphic effects in each of obese (rs4846914 tagging *GALNT2*) and lean (rs605066 tagging *CITED2*) populations as shown in Table 4.

In a second stage, we performed a sex-stratified analysis. The effect of neither weighted GRS_TG_ nor GRS_HDLc_ was found to be significantly different for males versus females for the population as a whole. Importantly, sexual dimorphism for genetic effects on HDLc was entirely driven by the obese subjects (*P_Interaction_* = 0.016) and was not evident in the lean (*P_Interaction_* = 0.914) or all (*P_Interaction_* = 0.0868) groups. Obese men showed an attenuated increase in HDLc in response to GRS_HDLc_ as compared with women (**Fig. 3**). Loci in each subpopulation (*CITED2* for obese and *GALNT2* for lean) were found to be dimorphic (Table 4). However, after correction for multiple testing, these remained only nominally significant (FDR <15%), thus requiring confirmation in additional populations.

In summary, we have created weighted GRSs for each of TG and HDLc based on loci identified by the Global Lipids Consortium and tested effects in separate large, well-defined obese and lean populations; thus, our results are without discovery bias. Neither GRS_TG_ nor GRS_HDLc_ showed an association with adiposity (BMI) per se. Here we demonstrate convincing gene-adiposity trait interactions. Notably, lean subjects have an ∼50% reduction in the genetic predisposition for increased TGs and an ∼35% greater response to HDLc-raising alleles, as compared with obese subjects. These effects are mainly driven by SNPs tagging *APOA5*,* GCKR*, and *LPL* for TG, and *CETP*,* LIPG*,* GALNT2*, and* PLTP* for HDLc. We also report sexual dimorphism for genetic effects on HDLc that is confined to the obese group of subjects. These findings demonstrate that obese individuals are more susceptible to genetic risk for dyslipidemia. SNP by BMI interactions may provide biological insight into specific genetic associations and missing heritability.

## Supplementary Material

Supplemental Data
