# Supplementary material for: Adiposity significantly modifies genetic risk for dyslipidemia
Source: J Lipid Res. 2014 Nov;55(11):2416–22. doi: 10.1194/jlr.P052522 (PMC4617143; doi:10.1194/jlr.P052522)
Supplement: Supplemental Data [file supp_55_11_2416__index.html]

Adiposity Significantly Modifies Genetic Risk for Dyslipidemia — Adiposity significantly modifies genetic risk for dyslipidemia — Supplemental Data 

# Adiposity significantly modifies genetic risk for dyslipidemia

## Supplemental Data

**Files in this Data Supplement:**

- Supplemental Information - Supplemental Table
