# Supplementary material for: Adiposity significantly modifies genetic risk for dyslipidemia
Source: J Lipid Res. 2014 Nov;55(11):2416–22. doi: 10.1194/jlr.P052522 (PMC4617143; doi:10.1194/jlr.P052522)
Supplement: Supplemental Data [file supp_P052522_jlr.P052522-1.doc]

**Supplementary Table 1: Effect sizes and loci used in the construction of the GRS.**

| **Lead Triglyceride SNPs** | | | | |
| --- | --- | --- | --- | --- |
| **Locus** | **Chromosome** | **Lead SNP** | **Minor Allele** | **Effect Size** |
| ANGPTL3 | 1 | rs2131925 | G | -4.94 |
| GCKR | 2 | rs1260326 | T | 8.76 |
| COBLL1 | 2 | rs10195252 | C | -2.01 |
| MSL2L1 | 3 | rs645040 | G | -2.22 |
| KLHL8 | 4 | rs442177 | G | -2.25 |
| MAP3K1 | 5 | rs9686661 | T | 2.57 |
| MLXIPL | 7 | rs17145738 | T | -9.32 |
| TYW1B | 7 | rs13238203 | T | -7.91 |
| TRIB1 | 8 | rs2954029 | T | -5.64 |
| T2 | 8 | rs1495741 | G | 2.85 |
| LPL | 8 | rs12678919 | G | -13.64 |
| PINX1 | 8 | rs11776767 | C | 2.01 |
| JMJD1C | 10 | rs10761731 | T | -2.38 |
| APOA1 | 11 | rs964184 | G | 16.95 |
| FADS1-2-3 | 11 | rs174546 | T | 3.82 |
| LRP1 | 12 | rs11613352 | T | -2.7 |
| FRMD5 | 15 | rs2929282 | T | 5.13 |
| CAPN3 | 15 | rs2412710 | A | 7 |
| CTF1 | 16 | rs11649653 | G | -2.13 |
|  |  | rs1042034 | C | -5.99 |
| **Lead HDLc SNPs** | | | | |
| GALNT2 | 1 | rs4846914 | G | 0.61 |
| PABPC4 | 1 | rs4660293 | G | 0.48 |
| ZNF648 | 1 | rs1689800 | G | 0.47 |
| IRS1 | 2 | rs2972146 | G | -0.46 |
| SLC39A8 | 4 | rs13107325 | T | 0.84 |
| ARL15 | 5 | rs6450176 | A | 0.49 |
| CITED2 | 6 | rs605066 | C | 0.39 |
| C6orf106 | 6 | rs2814944 | A | 0.49 |
| KLF14 | 7 | rs4731702 | T | -0.59 |
| PPP1R3B | 8 | rs9987289 | A | 1.21 |
| TRPS1 | 8 | rs2293889 | T | 0.44 |
| ABCA1 | 9 | rs1883025 | T | 0.94 |
| LRP4 | 11 | rs3136441 | C | -0.78 |
| AMPD3 | 11 | rs2923084 | G | 0.41 |
| SCARB1 | 12 | rs838880 | C | -0.61 |
| MVK | 12 | rs7134594 | C | 0.44 |
| ZNF664 | 12 | rs4765127 | T | -0.44 |
| SBNO1 | 12 | rs4759375 | T | -0.86 |
| LACTB | 15 | rs2652834 | A | 0.39 |
| LIPC | 15 | rs1532085 | A | -1.45 |
| CETP | 16 | rs3764261 | A | -3.39 |
| LCAT | 16 | rs16942887 | A | -1.27 |
| ABCA8 | 17 | rs4148008 | G | 0.42 |
| PGS1 | 17 | rs4129767 | G | 0.39 |
| STARD3 | 17 | rs11869286 | G | 0.48 |
| LIPG | 18 | rs7241918 | G | 1.31 |
| MC4R | 18 | rs12967135 | A | 0.42 |
| LOC55908 | 19 | rs737337 | C | 0.64 |
| ANGPTL4 | 19 | rs7255436 | C | 0.45 |
| LILRA3 | 19 | rs386000 | C | -0.83 |
| PLTP | 20 | rs6065906 | C | 0.93 |
| HNF4A | 20 | rs1800961 | T | 1.88 |
| UBE2L3 | 22 | rs181362 | T | 0.46 |
|  |  | rs12328675 | C | -0.68 |
| **Lead LDLc SNPs** | | | | |
| SORT1 | 1 | rs629301 | G | -5.65 |
| PCSK9 | 1 | rs2479409 | G | 2.01 |
| ABCG5/8 | 2 | rs4299376 | G | 2.75 |
| APOB | 2 | rs1367117 | A | 4.05 |
| MYLIP | 6 | rs3757354 | T | -1.43 |
| HFE | 6 | rs1800562 | A | -2.22 |
| LPA | 6 | rs1564348 | C | -0.56 |
| ST3GAL4 | 11 | rs11220462 | A | 1.95 |
| OSBPL7 | 17 | rs7206971 | A | 0.78 |
| APOE | 19 | rs4420638 | G | 7.14 |
| TOP1 | 20 | rs6029526 | A | 1.39 |

The gene listed in the ‘Locus’ column is either a plausible biological candidate gene in the locus or the nearest annotated.

**Supplementary Table 2 a) Interaction of obesity status with individuals SNPs in TG with 10% false discovery rate.** Genes identified in the text displayed lower P values than this threshold. **b) Interaction of obesity status with individuals SNPs in HDLc with 10% false discovery rate.** Genes identified in the text displayed lower P values than this threshold.

a)

| **Loci** | **SNP** | **Minor Allele** | **n** | **Interaction**  **Beta** | **Interaction**  **SE** | **P FDR** |
| --- | --- | --- | --- | --- | --- | --- |
| LPL | rs12678919 | G | 1877 | -0.2111 | 0.06216 | 5.00E-03 |
| APOA1 | rs964184 | G | 2360 | 0.1452 | 0.05544 | 1.00E-02 |
| GCKR | rs1260326 | T | 2758 | 0.1204 | 0.05483 | 1.50E-02 |
| TYW1B | rs13238203 | T | 130 | 0.4558 | 0.26 | 2.00E-02 |
| MSL2L1 | rs645040 | G | 2709 | 0.08513 | 0.05329 | 2.50E-02 |
| TRIB1 | rs2954029 | T | 2807 | -0.08454 | 0.05602 | 3.00E-02 |
| CTF1 | rs11649653 | G | 2709 | -0.0805 | 0.05507 | 3.50E-02 |
| COBLL1 | rs10195252 | C | 2804 | 0.06871 | 0.05531 | 4.00E-02 |
|  | rs1042034 | C | 2813 | -0.05433 | 0.05204 | 4.50E-02 |
| MLXIPL | rs17145738 | T | 2829 | -0.0527 | 0.05187 | 5.00E-02 |
| T2 | rs1495741 | G | 1889 | 0.06342 | 0.06482 | 5.50E-02 |
| KLHL8 | rs442177 | G | 2641 | -0.05455 | 0.0566 | 6.00E-02 |
| CAPN3 | rs2412710 | A | 2673 | -0.03539 | 0.05301 | 6.50E-02 |
| PINX1 | rs11776767 | C | 2019 | -0.03395 | 0.06401 | 7.00E-02 |
| LRP1 | rs11613352 | T | 2693 | 0.02335 | 0.05304 | 7.50E-02 |
| FRMD5 | rs2929282 | T | 2812 | 0.02022 | 0.05144 | 8.00E-02 |
| JMJD1C | rs10761731 | T | 2484 | -0.01925 | 0.05802 | 8.50E-02 |
| FADS1-2-3 | rs174546 | T | 2840 | 0.01652 | 0.05392 | 9.00E-02 |
| ANGPTL3 | rs2131925 | G | 2841 | 0.007925 | 0.05378 | 9.50E-02 |
| MAP3K1 | rs9686661 | T | 1075 | -0.00499 | 0.08792 | 1.00E-01 |

b)

| **Loci** | **SNP** | **Minor Allele** | **n** | **Interaction**  **Beta** | **Interaction**  **SE** | **P FDR** |
| --- | --- | --- | --- | --- | --- | --- |
| CETP | rs3764261 | A | 2415 | -0.2088 | 0.04747 | 2.94E-03 |
| GALNT2 | rs4846914 | G | 2829 | -0.1349 | 0.04545 | 5.88E-03 |
| LIPG | rs7241918 | G | 2329 | 0.1302 | 0.04822 | 8.82E-03 |
| PLTP | rs6065906 | C | 2769 | 0.1023 | 0.04423 | 1.18E-02 |
| LRP4 | rs3136441 | C | 2538 | -0.07974 | 0.04546 | 1.47E-02 |
| ZNF648 | rs1689800 | G | 2813 | 0.07529 | 0.04458 | 1.76E-02 |
| C6orf106 | rs2814944 | A | 2692 | -0.07186 | 0.04376 | 2.06E-02 |
| STARD3 | rs11869286 | G | 952 | -0.1126 | 0.06925 | 2.35E-02 |
| ARL15 | rs6450176 | A | 2806 | 0.05962 | 0.04207 | 2.65E-02 |
| ABCA8 | rs4148008 | G | 2321 | -0.06429 | 0.04853 | 2.94E-02 |
|  | rs12328675 | C | 2807 | -0.0534 | 0.04236 | 3.24E-02 |
| ANGPTL4 | rs7255436 | C | 135 | 0.2306 | 0.1833 | 3.53E-02 |
| PABPC4 | rs4660293 | G | 2824 | -0.04592 | 0.04398 | 3.82E-02 |
| LIPC | rs1532085 | A | 2279 | 0.04881 | 0.05179 | 4.12E-02 |
| KLF14 | rs4731702 | C | 2675 | 0.04375 | 0.0471 | 4.41E-02 |
| LCAT | rs16942887 | A | 2826 | -0.03597 | 0.04165 | 4.71E-02 |
| SLC39A8 | rs13107325 | T | 2357 | 0.03862 | 0.04839 | 5.00E-02 |
| SCARB1 | rs838880 | C | 2365 | -0.03746 | 0.05007 | 5.29E-02 |
| ZNF664 | rs4765127 | T | 2828 | -0.03101 | 0.04413 | 5.59E-02 |
| ABCA1 | rs1883025 | T | 2398 | 0.03144 | 0.0491 | 5.88E-02 |
| LILRA3 | rs386000 | C | 2352 | 0.02982 | 0.04763 | 6.18E-02 |
| HNF4A | rs1800961 | T | 2357 | 0.03002 | 0.04797 | 6.47E-02 |
| MC4R | rs12967135 | A | 2345 | -0.03164 | 0.05063 | 6.76E-02 |
| AMPD3 | rs2923084 | G | 1885 | 0.03158 | 0.05815 | 7.06E-02 |
| TRPS1 | rs2293889 | T | 2803 | -0.0224 | 0.04504 | 7.35E-02 |
| PGS1 | rs4129767 | A | 2319 | 0.02327 | 0.05202 | 7.65E-02 |
| CITED2 | rs605066 | C | 2684 | 0.01838 | 0.04601 | 7.94E-02 |
| IRS1 | rs2972146 | G | 2822 | -0.01659 | 0.0447 | 8.24E-02 |
| UBE2L3 | rs181362 | T | 2679 | -0.01614 | 0.04397 | 8.53E-02 |
| SBNO1 | rs4759375 | T | 2706 | 0.01511 | 0.04274 | 8.82E-02 |
| PPP1R3B | rs9987289 | A | 2816 | -0.01256 | 0.04066 | 9.12E-02 |
| LOC55908 | rs737337 | C | 597 | 0.00409 | 0.07695 | 9.41E-02 |
| MVK | rs7134594 | C | 2819 | -0.00208 | 0.04552 | 9.71E-02 |
| LACTB | rs2652834 | A | 2748 | 0.000629 | 0.04345 | 1.00E-01 |

**Supplementary Table 3: a) HDL Lean population Gene x Sex with 15% false discovery rate (FDR).** Genes identified in the text displayed lower P values than this threshold. **b) HDL Obese population Gene × Sex with 15% false discovery rate displayed.** Genes identified in the text displayed lower P values than this threshold.

a)

| **Loci** | **SNP** | **Minor Allele** | **n** | **Interaction Beta** | **Interaction SE** | **P FDR** |
| --- | --- | --- | --- | --- | --- | --- |
| CITED2 | rs605066 | C | 1409 | 0.1179 | 0.04152 | 4.84E-03 |
| SCARB1 | rs838880 | C | 1194 | -0.08932 | 0.04185 | 9.68E-03 |
| ARL15 | rs6450176 | A | 1451 | -0.06246 | 0.03532 | 1.45E-02 |
| ABCA1 | rs1883025 | T | 1233 | 0.06346 | 0.03877 | 1.94E-02 |
|  | rs12328675 | C | 1451 | -0.04849 | 0.03366 | 2.42E-02 |
| LACTB | rs2652834 | A | 1431 | 0.04818 | 0.0352 | 2.90E-02 |
| C6orf106 | rs2814944 | A | 1379 | -0.04266 | 0.03406 | 3.39E-02 |
| PGS1 | rs4129767 | G | 1173 | -0.05759 | 0.05253 | 3.87E-02 |
| PABPC4 | rs4660293 | G | 1460 | -0.03793 | 0.03594 | 4.35E-02 |
| TRPS1 | rs2293889 | T | 1448 | 0.04197 | 0.04181 | 4.84E-02 |
| LIPC | rs1532085 | A | 1184 | 0.04237 | 0.04336 | 5.32E-02 |
| PPP1R3B | rs9987289 | A | 1460 | -0.03132 | 0.03206 | 5.81E-02 |
| PLTP | rs6065906 | C | 1435 | 0.03508 | 0.03627 | 6.29E-02 |
| LCAT | rs16942887 | A | 1461 | -0.02881 | 0.0336 | 6.77E-02 |
| MVK | rs7134594 | C | 1458 | -0.0382 | 0.04456 | 7.26E-02 |
| ZNF648 | rs1689800 | G | 1452 | 0.03268 | 0.03988 | 7.74E-02 |
| AMPD3 | rs2923084 | G | 919 | 0.03489 | 0.04314 | 8.23E-02 |
| ZNF664 | rs4765127 | T | 1462 | -0.02568 | 0.03923 | 8.71E-02 |
| ABCA8 | rs4148008 | G | 1173 | 0.0245 | 0.04338 | 9.19E-02 |
| LILRA3 | rs386000 | C | 1188 | -0.01929 | 0.03799 | 9.68E-02 |
| GALNT2 | rs4846914 | G | 1463 | 0.0149 | 0.04077 | 1.02E-01 |
| UBE2L3 | rs181362 | T | 1370 | -0.01152 | 0.03574 | 1.06E-01 |
| HNF4A | rs1800961 | T | 1226 | -0.00996 | 0.03207 | 1.11E-01 |
| LIPG | rs7241918 | G | 1178 | 0.01058 | 0.0372 | 1.16E-01 |
| MC4R | rs12967135 | A | 1186 | -0.01057 | 0.0394 | 1.21E-01 |
| CETP | rs3764261 | A | 1238 | 0.01097 | 0.04139 | 1.26E-01 |
| IRS1 | rs2972146 | G | 1460 | -0.00803 | 0.03921 | 1.31E-01 |
| SBNO1 | rs4759375 | T | 1391 | 0.006367 | 0.035 | 1.35E-01 |
| KLF14 | rs4731702 | T | 1404 | 0.004652 | 0.04676 | 1.40E-01 |
| SLC39A8 | rs13107325 | T | 1226 | 0.001089 | 0.0346 | 1.45E-01 |
| LRP4 | rs3136441 | C | 1317 | 0.000869 | 0.03449 | 1.50E-01 |
| STARD3 | rs11869286 | G | 512 | NA | NA | 1.03E-01 |
| ANGPTL4 | rs7255436 | C | 50 | NA | NA | 1.06E-01 |
| LOC55908 | rs737337 | C | 317 | NA | NA | 1.10E-01 |

b)

| **Loci** | **SNP** | **Minor Allele** | **n** | **Interaction Beta** | **Interaction SE** | **P FDR** |
| --- | --- | --- | --- | --- | --- | --- |
| GALNT2 | rs4846914 | G | 1212 | 0.136 | 0.04411 | 4.84E-03 |
| LIPC | rs1532085 | A | 1022 | -0.07881 | 0.04612 | 6.45E-03 |
| CETP | rs3764261 | A | 1083 | -0.06724 | 0.04209 | 9.68E-03 |
| PLTP | rs6065906 | C | 1184 | 0.05309 | 0.03735 | 1.29E-02 |
| PPP1R3B | rs9987289 | A | 1204 | 0.04104 | 0.03334 | 1.61E-02 |
| ABCA1 | rs1883025 | T | 1071 | 0.0459 | 0.04145 | 1.94E-02 |
| ZNF664 | rs4765127 | T | 1212 | 0.04682 | 0.04274 | 2.26E-02 |
| LCAT | rs16942887 | A | 1211 | 0.03499 | 0.03387 | 2.58E-02 |
| UBE2L3 | rs181362 | T | 1170 | -0.03762 | 0.03724 | 2.90E-02 |
| ABCA8 | rs4148008 | G | 1060 | 0.0436 | 0.04385 | 3.23E-02 |
| LIPG | rs7241918 | G | 1061 | 0.03728 | 0.03859 | 3.55E-02 |
|  | rs12328675 | C | 1206 | -0.03109 | 0.03504 | 3.87E-02 |
| CITED2 | rs605066 | C | 1142 | 0.03903 | 0.04477 | 4.19E-02 |
| AMPD3 | rs2923084 | G | 945 | -0.03529 | 0.04198 | 4.52E-02 |
| C6orf106 | rs2814944 | A | 1172 | 0.02944 | 0.03595 | 4.84E-02 |
| SBNO1 | rs4759375 | T | 1169 | 0.02604 | 0.03587 | 5.16E-02 |
| MC4R | rs12967135 | A | 1069 | -0.02903 | 0.04081 | 5.48E-02 |
| LILRA3 | rs386000 | C | 1079 | 0.02757 | 0.04009 | 5.81E-02 |
| PGS1 | rs4129767 | A | 1058 | 0.03518 | 0.05163 | 6.13E-02 |
| SCARB1 | rs838880 | C | 1086 | 0.02741 | 0.04311 | 6.45E-02 |
| HNF4A | rs1800961 | T | 1054 | 0.02079 | 0.03501 | 6.77E-02 |
| TRPS1 | rs2293889 | T | 1204 | 0.02542 | 0.04436 | 7.10E-02 |
| KLF14 | rs4731702 | C | 1141 | 0.02457 | 0.05008 | 7.42E-02 |
| PABPC4 | rs4660293 | G | 1210 | -0.00549 | 0.03846 | 7.74E-02 |
| ZNF648 | rs1689800 | G | 1209 | 0.005909 | 0.04185 | 8.06E-02 |
| LRP4 | rs3136441 | C | 1103 | 0.004682 | 0.03617 | 8.39E-02 |
| IRS1 | rs2972146 | G | 1208 | 0.004336 | 0.04196 | 8.71E-02 |
| LACTB | rs2652834 | A | 1165 | 0.003484 | 0.03733 | 9.03E-02 |
| ARL15 | rs6450176 | A | 1202 | -0.00325 | 0.03726 | 9.35E-02 |
| MVK | rs7134594 | C | 1207 | -0.00328 | 0.0466 | 9.68E-02 |
| SLC39A8 | rs13107325 | T | 1054 | 8.19E-05 | 0.03537 | 1.00E-01 |
| STARD3 | rs11869286 | G | 299 | NA | NA | 1.03E-01 |
| ANGPTL4 | rs7255436 | C | 64 | NA | NA | 1.06E-01 |
| LOC55908 | rs737337 | C | 199 | NA | NA | 1.10E-01 |
